# Genome-wide phenotypic insights into mycobacterial virulence using *Drosophila melanogaster*

**DOI:** 10.1371/journal.ppat.1013474

**Published:** 2025-09-05

**Authors:** Niruja Sivakumar, Esther Fuentes, Linn-Karina Selvik, Marta Arch, Christina Gabrielsen Ås, Pere-Joan Cardona, Thomas R. Ioerger, Marte Singsås Dragset

**Affiliations:** 1 Centre for Molecular Inflammation Research (CEMIR), Norwegian University of, Science and Technology (NTNU), Trondheim, Norway; 2 Department of Clinical and Molecular Medicine, Norwegian University of Science and Technology (NTNU), Trondheim, Norway; 3 Tuberculosis Research Unit, Germans Trias i Pujol Research Institute (IGTP), Badalona, Spain; 4 Comparative Medicine and Bioimage Centre of Catalonia (CMCiB), Germans Trias i Pujol Research Institute (IGTP), Badalona, Spain; 5 Genetics and Microbiology Department, Universitat Autònoma de Barcelona, Barcelona, Spain; 6 Department of Medical Microbiology, St. Olavs University Hospital, Trondheim, Norway; 7 Centro de Investigación Biomédica en Red en Enfermedades Respiratorias (CIBERES), Instituto de Salud Carlos III (ISCIII), Madrid, Spain; 8 Microbiology Department, Laboratori Clínic Metropolitana Nord, Germans Trias i Pujol University Hospital, Badalona, Spain; 9 Department of Computer Science and Engineering, Texas A&M University, College Station, Texas, United States of America; National Institutes of Health, UNITED STATES OF AMERICA

## Abstract

*Drosophila melanogaster* (*Drosophila*) is one of the most extensively studied animal models we have, with a broad, advanced, and organized research community. Yet, *Drosophila* has barely been exploited to understand the underlying mechanisms of mycobacterial infections, which cause some of the deadliest infectious diseases humans are currently battling. Here, we identified mycobacterial genes required for the pathogen’s growth during *Drosophila* infection. Using *Mycobacterium marinum* (*Mmar*) to model mycobacterial pathogens, we first validated that an established mycobacterial virulence factor, EccB1 of the ESX-1 Type VII secretion system, is required for *Mmar* growth within the flies. Subsequently, we identified *Mmar* virulence genes in *Drosophila* in a high-throughput genome-wide phenotypic manner using transposon insertion sequencing. Of the 181 identified virulence genes, the vast majority (91%) had orthologs in the tuberculosis-causing *M. tuberculosis* (*Mtb*), suggesting that the encoded virulence mechanisms may be conserved across *Mmar* and *Mtb* species. By studying one of the identified genes in more depth, the putative ATP-binding protein ABC transporter encoded by *mmar_1660*, we found that both the *Mmar* gene and its *Mtb* ortholog (*rv3041c*) were required for virulence in human macrophages as well. We pinpointed the probable virulence mechanism of the genes to their requirements for growth during iron limitation, a condition met by mycobacteria during host infection. Together, our results bring forward *Drosophila* as a promising host model to study and identify mycobacterial virulence factors, providing insights that may transfer to *Mtb* human infection.

## Author summary

*Drosophila melanogaster* (*Drosophila*) has been fundamental to our knowledge of human biology, innate immunity included. Even so, a limited but growing number of research articles on the use of *Drosophila* or *Drosophila*-derived cell lines to understand mycobacterial infection biology have so far been published (17 to date). As such, *Drosophila* harness the exciting but hitherto untapped potential to fast-forward our understanding of underlying mechanisms of mycobacterial host-pathogen interactions, relevant for new antimycobacterial drug, vaccine, and diagnostic tool development. To further explore and expand *Drosophila*’s potential in mycobacterial research, we need genome-wide insights into the mycobacterial determinants causing disease in the fly. Here, we globally identified mycobacterial virulence factors at play during fly infection and showed that our novel findings may translate to a deeper understanding of the top-killer *Mycobacterium tuberculosis’* virulence mechanisms during human cell infection.

## Introduction

Tuberculosis (TB), caused by *Mycobacterium tuberculosis* (*Mtb*), accounted for 1.25 million deaths in 2023 alone [1]. Drug-sensitive TB is treated with a combination of four antibiotics over the course of several months [2]. Such a long and complex regimen can be difficult for patients to complete and for health professionals to follow up. Together, this can promote poor adherence, nurturing unfavorable patient outcomes and the development of drug resistant *Mtb* [2–4]. Novel strategies to improve treatment and reduce the development of *Mtb* resistance include targeting host-pathogen interactions (HPIs) pivotal for pathogen virulence and host immune responses [5–8]. HPIs are also highly relevant for new vaccine and diagnostic tool development [9–11]. Animal models to study HPIs are required to understand biological implications in a whole system setting. However, although they continue to be invaluable, the use of traditional laboratory animals is demanding in cost, labor, and space. Furthermore, poor reproducibility in animal models has been under scrutiny, and can partly be explained by inherent limitations in the framework, a relevant consideration to make for TB studies due to the heterogenous nature of the disease [12,13]. Hence, to accelerate our understanding of mycobacterial HPIs we could benefit from alternative but representative animal models that address or circumvent typical limitations, such as feasible numbers of individual animals, mutants and strains in an experimental setup. *Drosophila melanogaster* (*Drosophila*) may be particularly fit for this purpose [14,15].

*Drosophila,* also known as the common fruit fly, has been fundamental to our understanding of molecular mechanisms in human biology and disease, sharing 60% of its DNA with us [16]. *Drosophila*’s immunity largely depends on the phagocytosis of invading pathogens by plasmatocytes (macrophage-like cells), followed by activation of the Toll or Imd (for Immune deficiency) pathways for antimicrobial peptide production [17]. It was the identification of *Drosophila*’s Toll cascade that led to the characterization of human toll like receptors (TLRs) [18], reshaping our understanding of the human innate immune system. *Drosophila* do not possess adaptive immunity, creating the opportunity to specifically study the innate immune responses in an isolated yet *in vivo* setting. Being a workhorse of basic biological research, the *Drosophila* scientific community is both advanced and open, with state-of-the-art molecular tools and fly mutants shared in an organized manner and at low cost. Importantly, *Drosophila* as a host model is in compliance with the principles of the 3Rs (Replace, Reduce, Refine), part of the European Commission legislation Directive 2010/63/EU, for a more humane animal research [19]. Strengthened by its short generation time and general ease to handle, the above makes *Drosophila* a powerful animal model to study innate immune responses to infection.

During infection of the human host, *Mtb* is taken up by phagocytotic macrophages and is able to replicate within them primarily by blocking acidification of phagosomes and their fusion to lysosomes [20]. Likewise, Dionne *et al.* showed that *M. marinum* (*Mmar*) proliferates within *Drosophila* plasmatocytes during infection, blocking acidification of the cells’ phagosomes [21]. *Mmar*, which mainly causes fish TB and skin infections in humans [22–24], thrives at 29°C like *Drosophila*, and is thus compatible with the fruit fly in an infection setting. In fact, we and others have used the *Drosophila* infection model to unravel how aspects of the fly’s immune response and reproductive status interact with mycobacteria during infection [14,25–31]. *Drosophila* has also been useful to assess antimycobacterial drug activity, infecting the fly with either *Mmar* or the clinically relevant fast-growing *M. abscessus* [32,33]. Recently, the use of *Drosophila* in mycobacterial research was summarized and reviewed, emphasizing *Drosophila*’s untapped potential to study mycobacterial HPIs [14]. To fully exploit *Drosophila* for this purpose, we need a deeper insight into the mycobacterial virulence-driving mechanisms at play during *Drosophila* infection.

In this study, we define virulence-causing genes as those required for the bacteria to fully propagate within the animal host but not on standard laboratory solid growth medium. Using *Mmar* as a model we identified 181 virulence genes in *Drosophila* in a genome-wide transposon insertion sequencing (TnSeq) screen. Interestingly, 91% of the genes had orthologs in *Mtb*, underlining the potential relevance of the *Mmar*-*Drosophila* infection model to study *Mtb* virulence. Finally, we knocked out a hit in our screen (*mmar_1660*) and its ortholog in *Mtb* (*rv3041c*) and demonstrated that the mutants were growth-impaired within human macrophages as well as under *in vitro* iron limited conditions. The latter points to a role for these genes in iron uptake, a virulence mechanism that enables *Mtb* to replicate in the low iron environment it encounters during infection of the human host [34,35].

## Results

### The established mycobacterial virulence factor EccB1 is required for *Mmar* virulence in *Drosophila.*

**Fig 1:**
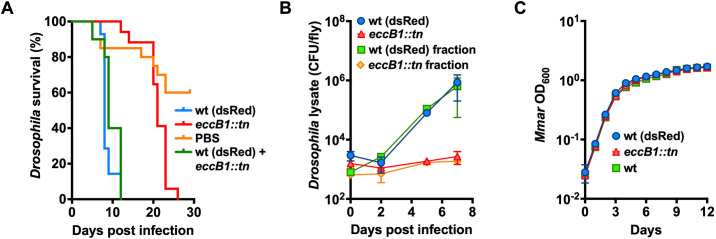
EccB1 is required for *Mmar* virulence in *Drosophila.* Male *Drosophila* infected with 5000 CFU/fly of *Mmar* E11 strain wt (dsRed), *eccB1::tn*, a 1:1 mix of wt (dsRed) and *eccB1::tn*, or PBS. Fly survival (A) and CFU per fly (B) was recorded over the course of infection. For fly survival **(A)**, data represent the percent survival of initial 30 flies per condition and were recorded daily. For CFU measurements **(B)**, wt (dsRed) and *eccB1::tn* were selected on hygromycin or kanamycin, respectively, and data represent means ± standard deviation (SD) for three individual flies per condition and time point. **(C)** *Mmar* E11 wt, wt (dsRed), and *eccB1::tn* growth *in vitro* (7H9 medium) as measured by OD_600_. Data represent means ± SD of three technical replica recorded every 24 hours.

For *Drosophila* to be a relevant host model to study mycobacterial virulence, established mycobacterial virulence factors should be required for *Mmar* infection in the fly. Hence, we infected *Drosophila* with an *Mmar* (strain E11) mutant in EccB1. EccB1 (encoded by *eccB1*) is a core component of the 6 kDa early secretory antigenic target (ESAT-6) Type VII secretion system 1 (ESX-1) [36]. ESX-1 is a hallmark of *Mtb* virulence through secretion of specific protein substrates, such as CFP-10 and ESAT-6, across the complex mycobacterial cell wall [37]. Indeed, we showed that disruption of *Mmar eccB1* (transposon insertion mutation; *eccB1::tn*) clearly increased the survival of flies upon infection, compared to *Mmar* wild type (wt) infection (Fig 1A). Correspondingly, the *eccB1* mutant showed attenuated growth within the flies (Fig 1B), most probably explaining the prolonged survival of the mutant-infected flies. The wt and *eccB1::tn* mutant strains grew comparably *in vitro* in 7H9 medium (Fig 1C). Hence, we confirmed the attenuated phenotype of an ESX-1-disrupted mutant during *Mmar Drosophila* infection.

### *Drosophila* is suitable for genome-wide screening for mycobacterial virulence genes

Next, we sought to determine whether *Drosophila* is suitable to identify novel mycobacterial virulence genes. By TnSeq, we and others have previously identified mycobacterial virulence genes in a genome-wide manner using mice [38–41], cattle [42], or amoeba [43–45], as host infection models. Discovery of virulence genes by TnSeq is based on infecting the host with a bacterial high-density transposon (tn) mutant library, followed by harvesting the library for analysis after infection, comparing its mutant constituents to the input library using massive parallel sequencing [46]. Genes with tn mutations that cause bacterial growth-impairment within the host but not on standard agar medium, are here defined as virulence genes. Hypothetically, a mutant deficient in for instance ESX-1 secretion could be rescued by mutants sufficient in ESX-1 secretion residing within the same plasmatocyte. To investigate whether this could obscure our screen for virulence genes when we pass a high number of mutants through one single fly, we infected *Drosophila* with 5000 colony forming units (CFU) per fly of either *Mmar* wt, *eccB1::tn* mutant or a 1:1 mix of *Mmar* wt and *eccB1::tn* mutant. By using an *Mmar* wt strain carrying a plasmid conferring hygromycin resistance and dsRed expression, wt (dsRed), in combination with the *eccB::tn* mutant carrying kanamycin resistance encoded within the tn insert, we could by antibiotic selection separate the growth of the mutant from wt growth during co-infection. As seen in Fig 1B, the *eccB1* mutant was still growth-attenuated within the flies when co-infected with *Mmar* wt, while the co-infected flies died similarly to those infected by wt only (Fig 1A). All strains grew comparably in 7H9 liquid medium (Fig 1C). From this, we take that virulence mutants are not necessarily rescued by co-infection with other mutants intact in their version of the virulence gene in question, although this may indeed vary depending on the mechanism of the gene. By day seven post infection the wt bacteria had doubled approximately seven times, enabling us to differentiate between mutants in virulence and non-virulence genes during TnSeq screening (Fig 1A). Taken together, our results suggest that *Drosophila* is a suitable host model to screen for mycobacterial virulence genes.

### Genome-wide identification of *Mmar* virulence genes in *Drosophila*

We aimed to identify *Mmar* virulence genes in *Drosophila*. Hence, we constructed and sequenced a high-density tn insertion library in the *Mmar* E11 strain using ϕMycoMarT7 [47]. The obtained library contained mutants in 80% of TA sites (ϕMycoMarT7 insertion sites) covering 96.5% of the genes. We uncovered 430 essential (ES) genes, 130 genes conferring growth defect (GD) when disrupted, 4314 non-essential (NE) genes, and 79 genes conferring growth-advantage (GA) when disrupted (S1A Dataset). Combining ES and GD categories, the number of 560 genes that are essential or cause a growth-defect when disrupted, is in line with what has been observed in *Mtb* [48,49]. In a previous TnSeq study of the *Mmar* E11 strain [44], 300 genes were identified as being essential *in vitro*, of which 82% (247) were also in the ES or GD category in our data (S1A Dataset).

To specifically identify *Mmar* virulence genes during *Drosophila* infection, we passed the generated library through *Drosophila* (250 male or female flies, 5000 CFU/fly), covering the mutant library 17 times across the 250 flies. We subjected the input and output libraries to TnSeq, the latter harvested from the flies seven days post infection. To show reproducibility when spreading the tn library across several hosts, we created three scatter plot matrices showing high correlation of the gene-level mean tn insertion counts among the three biological replicates of each condition *(in vitro* input library and *in vivo* output libraries passed through male or female flies)*.* The correlations in these plots demonstrate similar estimates of gene requirement across experimental replicates, suggesting that to spread the library over several hosts has no negative impact on the downstream analysis and result (S1 Fig).

**Fig 2:**
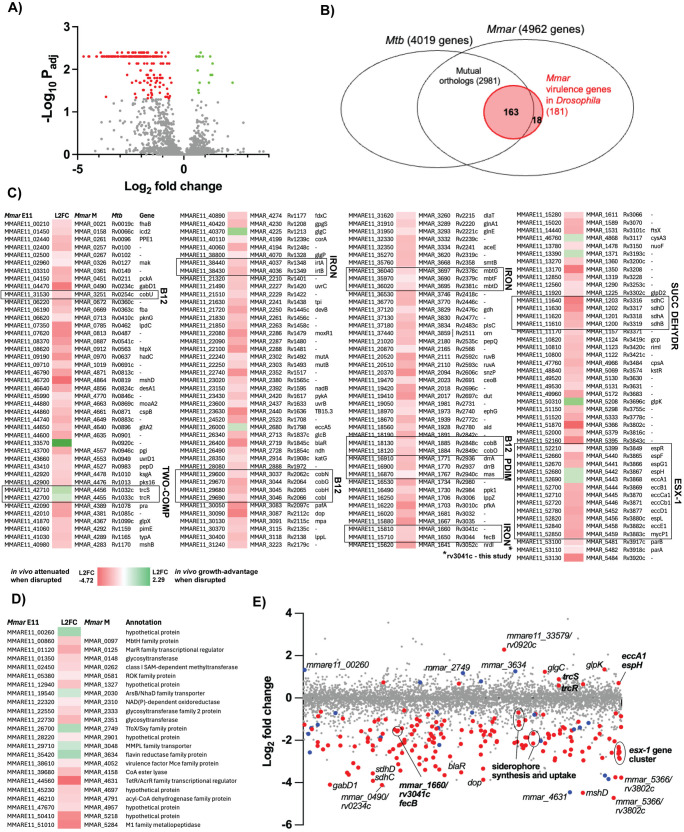
Genome-wide identification of *Mmar* virulence genes in *Drosophila.* (A) Volcano plot of *Mmar* E11 virulence genes in male *Drosophila* based on three independent experiments of 250 flies infected with 5000 CFU/fly of the *Mmar* E11 tn mutant library. The Log_2_ fold change indicates fold change of the mean normalized tn insertion counts per gene of *Mmar* selected *in* *vitro* (7H9) versus *in vivo* (after *Drosophila* infection). Each dot represents one gene. Dots in color represent genes with an adjusted P-value (using Benjamini-Hochberg correction, FDR < 0.05) <0.05. Red dots represent genes that are *in vivo* attenuated when disrupted (e.g., virulence genes), while green dots represent genes that confer *in vivo* growth advantage when disrupted. (B) Venn diagram illustrating *Mmar* virulence genes in *Drosophila* (red) overlayed the entire pool of *Mmar* (E11 strain) and *Mtb* (H37Rv strain) genes and their mutual orthologs. (C) List of *Mmar* E11 genes with adjusted P-value <0.05 that have *Mtb* mutual orthologs. Selected pathways are highlighted with black frames, IRON; genes involved in low iron growth, ESX-1; genes involved in ESX-1 secretion, B12; genes involved in cobalamin biosynthesis, PDIM; genes involved in PDIM synthesis, SUCC DEHYDR; genes encoding succinate dehydrogenase, TWO COMP; two-component signal transduction system. (D) List of *Mmar* E11 genes with adjusted P-value <0.05 but no *Mtb* mutual orthologs. (E) Plot of each *Mmar* E11 gene’s Log_2_ fold change respective to genomic position (from *mmare11_00010* to *mmare11_53170*). Dots in red represent *Mmar* E11 genes with an adjusted P-value <0.05 and *Mtb* orthologs, while blue dots represent *Mmar* E11 genes with an adjusted P-value <0.05 but no *Mtb* mutual ortholog. Genes that are either discussed in this study or have particularly high/low Log_2_ fold ratios are highlighted.

We found that 181 genes were required for optimal growth within the flies, based on a permutation test (“resampling analysis” [50]) of the difference in mean tn insertion counts per gene in libraries that had undergone fly infection (output) versus libraries grown under *in vitro* condition (input) (Log_2_ fold change <0 and adjusted P-value <0.05) (S1B Dataset and Fig 2A–E). Among the virulence genes identified were those encoding established mycobacterial virulence factors, such as by phthiocerol dimycoceroserate (PDIM, a cell wall lipid, *mmar_1767, _1770–1771*), components of the ESX-1 secretion system (10 genes between *mmar_5399–5459*), and the LytR-CpsA-Psr domain-containing protein CpsA (*mmar_4966*) (Fig 2C). Moreover, all the four genes encoding the succinate dehydrogenase Sdh2 (*mmar_1200–1203*) as well as seven genes involved in cobalamin (vitamin B12) biosynthesis were hits in our screen (Fig 2C). We also identified genes (15) that conferred *Mmar* growth advantage within *Drosophila* when disrupted (Log_2_ fold change >0 and adjusted P value <0.05), making them the mere opposite of virulence genes (S1B Dataset and Fig 2A, 2C–2E). Examples of these are ESX-1’s *eccA1* (*mmar_5443*) and *espH* (*mmar_5442*), and the two-component signal transduction system *trcS*/*trcR* (*mmar_4455–4456*) (Fig 2C). When we compared the virulence gene set of male versus female flies, we found three genes that were required for virulence in males only (S1C Dataset). Of these, two did not have *Mtb* orthologs, while the third encodes the NADH-quinone oxidoreductase subunit NuoF. Taken together, we identified 181 genes as required for full *Mmar* virulence during *Drosophila* infection, and 15 genes that conferred a growth advantage in the fly when disrupted.

### The relevance of *Mmar Drosophila* virulence to *Mtb* infection

To investigate the potential relevance of the *Mmar* virulence genes in *Drosophila* to *Mtb* infection, we compared them to the entire *Mtb* gene pool (Fig 2B). There are 2981 mutual orthologs shared between *Mmar* E11 and *Mtb* H37Rv, where a mutual ortholog here means that the gene in one organism is the best match for the ortholog in the other organism and vice versa, with a BLAST E value of <10^−10^. An unbiased proportion of *Mmar* virulence genes with *Mtb* mutual orthologs would be ~ 60%, while we found that that 91% (163/181 genes) had mutual orthologs in *Mtb* (Fig 2B–E). Moreover, when we compared the *Mmar* virulence genes to those previously identified by TnSeq for *Mtb*, 48% had *Mtb* orthologs that were defined as required for full *in vivo* growth in mice [39,41] (S1B Dataset). Together, our findings suggest that mechanisms of the identified virulence genes could be conserved across *Mmar* and *Mtb* and across the host model species in question.

### *mmar_1660* is a novel mycobacterial virulence gene

**Fig 3:**
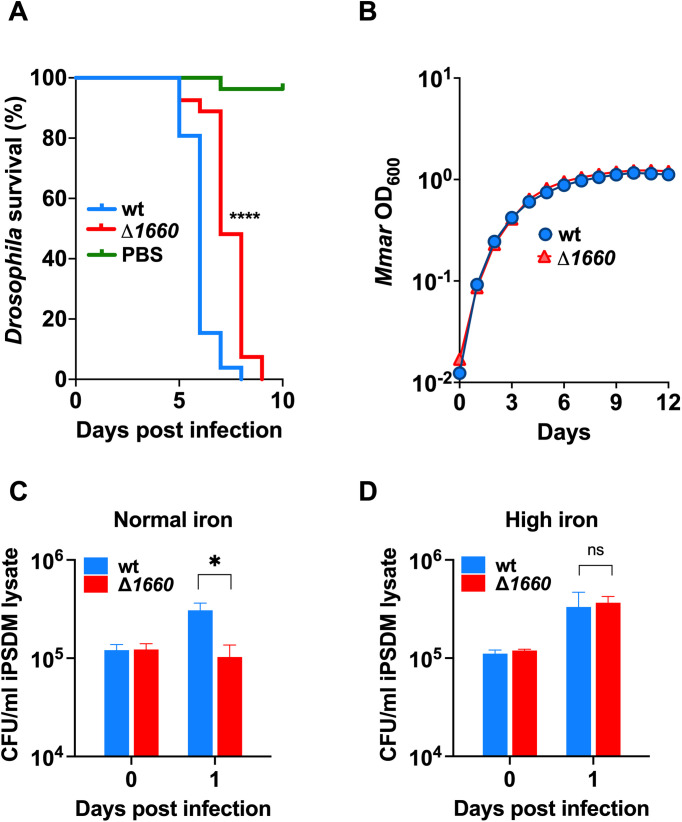
*mmar_1660* is required for full *Mmar* virulence in *Drosophila* and human macrophages. (A) Male *Drosophila* infected with 500 CFU/fly of *Mmar* M strain wt, Δ*1660* or PBS, with their survival recorded over the course of infection. Data represent the percent survival of initial 30 flies per condition and were recorded daily. The four asterisks represent a statistically significant difference with a P value <0.0001 between wt and Δ*1660*-infected flies as calculated by Log-rank Mantel-Cox testing using GraphPad Prism 9. (B) *Mmar* M wt and Δ*1660* growth *in vitro* (7H9 medium). Data represent means ± SD of three technical replicates recorded every 24 hours. Human iPSDMs were infected with *Mmar* M strain wt or Δ*1660* at a multiple of infection of 4:1 (macrophage:bacterium) under normal cell culture conditions (C) or with 100 μM FeAC added to the cell culture medium (D). C and D represent the combined averages of three technical triplicates from three independent experiments. Statistically significant difference was calculated using the Mann Whitney U-test on the datasets of day one post infection (unpaired, one-tailed). ns, not significant. *, P-value ≤ 0.05.

To validate our screen, we aimed to create a targeted knockout mutation of one of the virulence genes identified. Due to our previous experience and interest in mycobacterial low iron growth [51–53], we chose *mmar_1660* which encodes a putative conserved ATP-binding protein ABC transporter whose *Mtb* ortholog is suggested by homology to be involved in metal transport [54,55] and specifically transport of iron [56–58]. For reasons unknown we were unable to mutagenize the *Mmar* E11 strain in a targeted manner using bacteriophage-mediated allelic exchange, while we succeeded in the *Mmar* M strain. Hence, we infected *Drosophila* with 500 CFU/fly *Mmar* M wt or Δ*1660*, and found that Δ*1660*-infected flies survived on average one day longer than wt-infected flies (Fig 3A). The strains grew comparably *in vitro* in standard liquid medium (Fig 3B). The *Mmar* M strain has previously been reported to grow faster than the E11 strain under hypoxic conditions [59]. In line with this, we observed that the M strain killed *Drosophila* faster than the E11 strain by an average death after 6 days of 500 CFU/fly M strain infection compared to average death after 8 days of 5000 CFU/fly E11 infection (Figs 1A, 3A). Our screen showed that *mmar_1660* is among the genes giving rise to a moderate virulence phenotype when disrupted, with a Log_2_ fold change of -1.54 (Figs 2B, 2E). Thus, the modest but strongly significant one-day *in vivo* attenuation of the M strain Δ*1660* mutant’s growth (Fig 3A) in comparison to the 13-day attenuation of the E11 *eccB1* mutant (Fig 1A) may be due to an inherent faster growth rate of the M strain – shortening the window to detect virulence phenotypes – in combination with a less prominent role of *mmar_1660* during infection. Even so, these results validate *mmar_1660* as a virulence gene during *Drosophila* infection, a trait that transfers from the E11 to the M strain.

Furthermore, we wanted to investigate whether *mmar_1660* contributed to virulence in human macrophages. We infected human macrophages derived from induced pluripotent stem cell-derived macrophages (iPSDMs) with the *Mmar* M wt and Δ*1660* strain, and found that at day one post infection the wt but not the Δ*1660*-mutant had proliferated within the macrophages, indicating that the mutant is growth-impaired within the cells (Fig 3C). In summary, we show that *mmar_1660* is required for full virulence in *Drosophila* as well as in human macrophages.

### *mmar_1660* and its *Mtb* ortholog *rv3041c* are required for low iron growth

**Fig 4:**
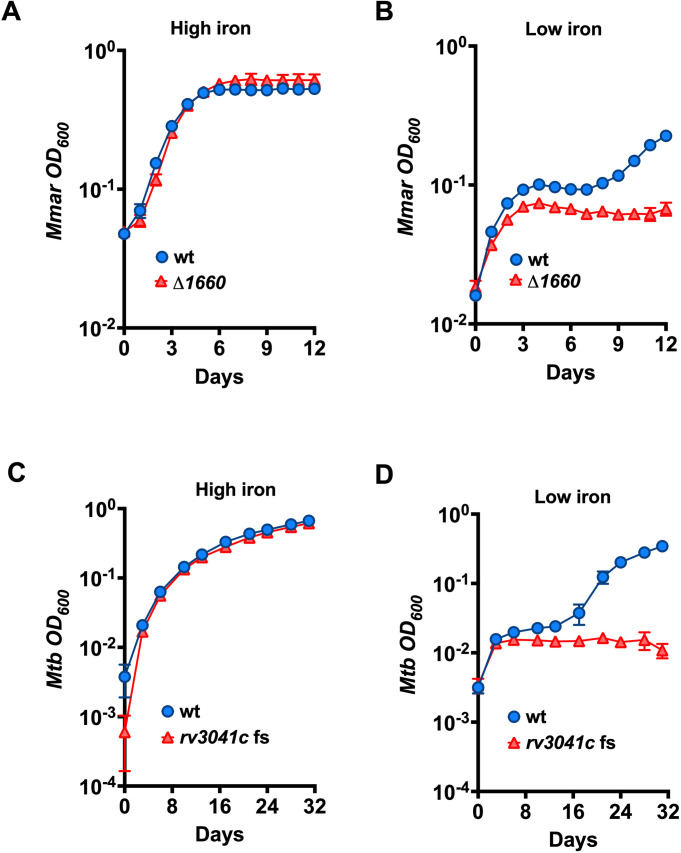
The orthologs *mmar_1660* and *rv3041c* are required for low iron growth. *Mmar* M (A, B) and *Mtb* (C, D) strains were grown in high (150 μM or 300 μM FeCl_3_, respectively) or low iron media as indicated. *Mtb* low iron media contained 1 μM DFO. Data represent means ± SD of three technical replicates of which OD_600_ was recorded regularly at the time points indicated in the figure.

*mmar_1660’s Mtb* ortholog *rv3041c* encodes a conserved ATP-binding ABC transporter of unknown function suggested to be involved iron transport [54–58]. To test whether these genes are indeed required for growth under iron limitations, we created a frameshift (fs) mutant causing a premature stop codon within *Mtb rv3041c* and subjected the Δ*1660* and the *rv3041c* fs mutants to growth in low iron *in vitro*. Both mutants showed impaired growth when iron was low, compared to their respective wt strains, while under high iron conditions (i.e., same level as in 7H9) they grew similarly to their respective wts (Fig 4A–D). The initial rise in OD_600_ values within the first 3 days in low iron medium is likely due to bacteria sedimenting at the bottom of the 96- or 100-well (honeycomb) plates used for *Mtb* and *Mmar* incubation, respectively, rather than actual bacterial growth.

To support the notion that the observed phenotypes were caused by the introduced mutations, we whole genome sequenced the strains. We successfully identified the introduced deletions in both *mmar_1660* and *rv3041c* (S2 Dataset). In addition to the introduced *rv3041c* fs mutation, we identified in this strain two single nucleotide polymorphisms (SNP) that distinguished the strain from the wt, creating an amino acid change in *rv2217* (*lipB*, amino acid 51 of 230 mutated from alanine to glutamic acid) and *rv3452* (*cut4*, amino acid 80 of 226 mutated from asparagine to threonine). Similar mutations were not found in the orthologous genes (or any other genes) in the *Mmar* Δ*1660* strain, strongly reducing the probability that one or both of the additional mutations contributed to the observed low iron phenotype in *Mtb*. Together, our results show that *mmar_1660* and its *Mtb* ortholog *rv3041c* are required for mycobacterial growth when iron availability is low.

### *mmar_1660* and *rv3041c* are required for virulence in human macrophages in an iron-dependent manner

**Fig 5:**
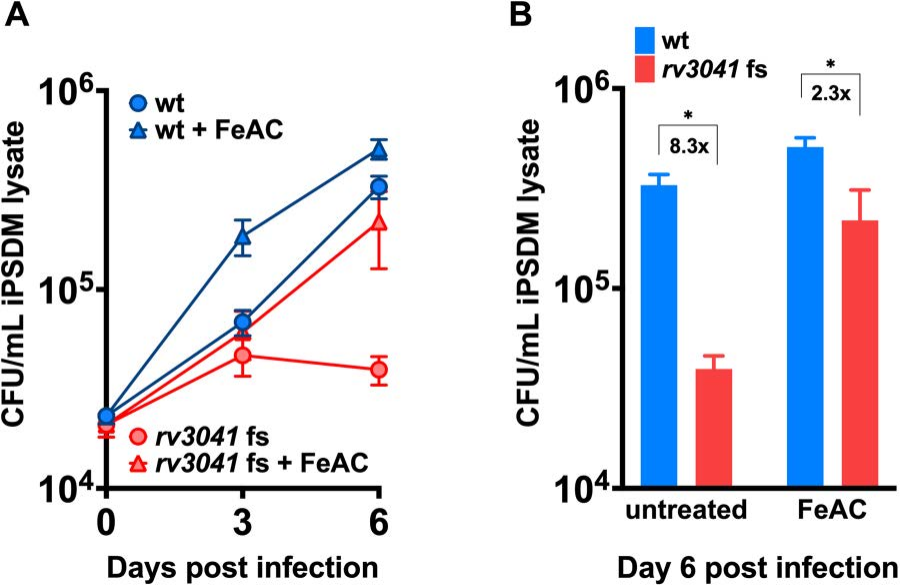
*rv3041c* is required for full virulence in human macrophages in an iron-dependent manner. Human iPSDMs were infected with *Mtb* wt or *rv3041c* fs strain at a multiple of infection of 2:1 (macrophage:bacterium). After 2-hour infection medium was supplemented with either 100 µM FeAC or no supplementation. (A) Intracellular bacterial burden was assessed by CFU enumeration of cell lysates at the given time points post infection. Data represents means ± SD from three technical replicates per condition. The data is representative of two (for FeAC) or three (for untreated) independent experiments (B) The relative growth differences between wt and *rv3041c* fs in the different conditions were determined on day 6 post infection. 8.3x and 2.3x indicate the fold difference between wt and *rv3041c* fs in unsupplemented (normal iron) and 100 μM FeAC supplemented (high iron), respectively. Statistically significant difference was calculated using the Mann Whitney U-test on the datasets of day 6 post infection (unpaired, one-tailed). *, P-value ≤ 0.05.

Iron acquisition is critical for survival and replication of *Mtb* in phagosomal containment of macrophages during infection [34,35]. Hence, to investigate whether *rv3041c* is required for *Mtb* virulence, we infected iPSDMs with the *rv3041c* fs and wt strains and found that the fs mutant was growth-impaired within the cells compared to the wt (Fig 5A-B). The role of iron in mycobacterial infections has been studied by others by supplementing iron to *in vitro* cell culture models [60–62]. When we applied the same principle to our *Mmar* and *Mtb* iPSDM infection models, we found that the *Mmar* Δ*1660* mutant’s intracellularly attenuated phenotype was fully rescued (Fig 3D) while the orthologous *rv3041c* fs mutant´s phenotype was partly rescued in macrophages cultivated in the presence of additional iron supplement (ferric ammonium citrate, FeAC) (Fig 5A). For *rv3041c*, this was evident when comparing the fold difference in CFU/ml lysate between normal and high iron conditions at day 6 post infection, where under standard conditions the fold difference between wt and fs mutant was 8.3 compared to only 2.3 when FeAC was added (Fig 5B). These results demonstrate that *mmar_1660* and *rv3041c* are less required for intra-macrophagic growth when iron is plentiful. In summary, *mmar_1660* and *rv3041c* are required for full virulence in human macrophages in a manner that depends on the host cell’s iron status.

## Discussion

*Drosophila* has been fundamental to our understanding of mechanisms underlying human biology, the response to infection included [17,18]. While *Drosophila* has previously been proved useful to understand host responses towards mycobacterial infections [14,25,27,28], we here show that it is too suitable to study mycobacterial infections from the pathogen perspective. Recently, a genome-wide association study of *M. abscessus* in combination with *in vivo* phenotyping, computational structural modelling, and epistatic analysis, predicted genes involved in *M. abscessus* virulence [26]. Three of the predicted genes were *in vivo* validated as required for virulence using *Drosophila* [26]. Another recent study identified an *M. abscessus* asparagine transporter required for full virulence in *Drosophila* [29]. Importantly, these studies too emphasize the applicability of *Drosophila* to research clinically important mycobacterial virulence.

By TnSeq of *Mmar* tn libraries before and after *Drosophila* infection, we found 181 genes that were required for full *Mmar* virulence. Of these, many were already established mycobacterial virulence genes, while others were novel (45 and 55%, respectively, based on *Mtb* mouse model TnSeq *in vivo* growth screening [39,41] (S1B Dataset). Of the identified *Mmar* virulence genes, 91% had orthologs in *Mtb*, possibly reflecting that certain virulence mechanisms relevant to innate immunity and growth within the macrophage/plasmatocyte may be conserved across *Mmar* and *Mtb*, and across arthropod (*Drosophila*) and vertebrate (mouse) host models. We recently found that sex and reproductive status of *Drosophila* influences its susceptibility to *Mmar* infection [25]. Even so, only three *Mmar* genes were differently required for virulence in male and female flies in our screens, suggesting that the *Mmar* virulence mechanisms at play during *Drosophila* infection depend minimally on the sex of the fly.

Among the already established mycobacterial virulence genes identified in our screen were those encoding factors involved in macrophage intracellular survival and escape. For instance, we found genes encoding PDIM which is thought to contribute to phagosomal escape and macrophage exit [63], and the ESX-1 secretion system which is thought to secrete immune modulating effectors and to facilitate phagosomal escape [64]. These findings are in line with previous descriptions by Dionne *et al.* of initial proliferation of *Mmar* within plasmatocytes before dissemination of the phagocytosed *Mmar* at later stages of *Drosophila* infection [21]. Touré et al. recently showed that *M. abscessus* resists killing by antimicrobial peptides and that they also reside within the flies’ plasmatocytes in early but not later stages of *Drosophila* infection [27,28]. They pointed out the role of thanacytes, another *Drosophila* immune cell population, in bacterial dissemination where thanacytes induced a caspase-dependent apoptotic death of the infected phagocytes [28]. A similar mechanism may be at play during *Mmar Drosophila* infection.

Interestingly, all four genes encoding succinate dehydrogenase Sdh2 were required for full *Mmar* virulence within the fly. *Mtb* (and *Mmar* E11) encodes two succinate dehydrogenases, Sdh1 (*rv0247*–*0249*) and Sdh2 (*rv3316*–*3319*), where the two were found to have redundant functions during *Mtb* growth on a variety of carbon sources [65]. While Sdh1- and partly Sdh2-encoding genes were defined as required for *in vivo* growth during *Mtb* mouse infection (according to *Mtb* TnSeq screening [39,41]) (S1B Dataset), *Mmar* might not require the Sdh1 succinate dehydrogenase for full virulence in the fly, as none of the Shd1-encoding genes were hits in our screen. Together, this may shed light on non-redundant roles of the two enzymes during host infection.

Among genes that were required for full *Mmar Drosophila* virulence, but not identified as required for *Mtb in vivo* growth in mice, were those encoding cobalamin (vitamin B12) biosynthesis. *Mtb* depends on the uptake of cobalamin during mouse infection, and is not able to produce the vitamin intracellularly [66]. In contrast, cobalamin is synthesised by non-tuberculous mycobacteria [67]. Both the *Mmar* M and E11 strains contain mutual orthologs of the *Mtb* cobalamin transporter BacA, *rv1819c* (S1B Dataset) [68]. The *Mmar* M strain has been found to extracellularly scavenge cobalamin, but although the vitamin is available to the pathogen during zebrafish embryo infection, a mutant in cobalamin synthesis was attenuated within the fish [69]. Cobalamin biosyntheis was also required for the growth of *Mmar* M during *Dictyostelium discoideum* amoeba infection, but curiously conferred a growth-advantage to *Mmar* E11 when disrupted during *Acanthamoeba castellanii* amoeba infection [44,70]. Even so, while *Mmar* E11 may be able to scavenge cobalamin from *Drosophila* during infection, it relies on cobalamin biosynthesis for full virulence in the fly.

Among the *Mmar* genes that mediated a growth advantage within *Drosophila* when disrupted, were *eccA1* and *espH* of the ESX-1 secretion system, and the two-component signal transduction system *trcS*/*trcR*. EccA1 is thought to regulate mycolic acid lipid synthesis [71], in addition to facilitate ESX-1-mediated secretion of the key mycobacterial virulence factors ESAT-6 and CFP-10 [72]. Weerdenburg *et al.* found that in cell culture, EccA1 was required for virulence in mammalian but not in protozoan cells [44]. *trcS*/*trsR* regulates the expression of *rv1057*, encoding a seven-bladed β-propeller [73]. Interestingly, an *rv1057* deletion mutant reduced ESAT-6 secretion and *Mtb* intracellular growth within human macrophages [74]. EccA1 and TrcS/TrcR may therefore be involved in fine-tuning ESAT-6 secretion and modulate the interaction between the pathogen and the macrophage, and in certain host species their absence may lead to a growth advantage, perhaps by removing functional ESX-1 secretion brakes. For *espH*, Pahn *et al*. described a role of the encoded protein in the secretion of ESX-1 substrates EspE and EspF, potentially as a specific chaperone [75]. They too observed the hypervirulent phenotype of an *espH* deletion mutant in zebrafish larvae, but not during *in vitro* mouse macrophages or *A. castellanii* amoeba infection, speculating that *espH* is required for an ESX-1 secretion-dependent homeostatic balance between host and pathogen during granuloma formation. A similar mechanism of *espH* may be at play during *Mmar Drosophila* infection, although not in a granuloma-specific fashion.

We validated *mmar_1660* as required for full virulence during *Drosophila* infection, and both *mmar_1660* and its *Mtb* ortholog *rv3041c* as required for full virulence in human macrophages. This is in line with published TnSeq screens, defining *rv3041c* as a virulence gene in C57BL/6 [41], IFNγ knockout, and collaborative cross 001 and 027 mouse strains [39], as well as required for growth under hypoxic condition, mimicking an infection setting [76]. *mmar_1660* and *rv3041c* encode putative ATP-binding proteins of ABC transporters [58], predicted to be involved in active transport of iron across the membrane [56,57]. Iron is an essential nutrient for most organisms, including mycobacteria which rely on various strategies like siderophore and hemophore production to scavenge ferric iron and heme, respectively [35,77]. By mutating *mmar_1660* and *rv3041c,* we confirmed that the genes were indeed required for growth under *in vitro* iron limited conditions and for virulence in human macrophages in a manner that depended on the host cell’s iron status. The genes may therefore be involved in a novel mechanism to obtain iron during infection, encode hitherto unknown components of already known iron uptake pathways, or by other means be involved in low iron growth. It cannot, however, be excluded that *mmar_1660* has another function, unrelated to iron uptake, during *Drosophila* infection. With that said, *Mmar* orthologs of genes known to be involved in *Mtb* (carboxy)mycobactin siderophore import (*irtA, irtB*) and biosynthesis (*mbtD, mbtF, mbtG*) [78,79], were defined as virulence genes in our screen with similar Log_2_ fold changes to *mmar_1660* (Fig 2E). *mmar_1660* and *rv3041c* are seemingly the respective first genes in a two or three-gene operon, with the other two genes being a conserved hypothetical and probable enoyl-CoA hydratase *echA17* [58]. While it is not clear which if any role the putatively co-expressed genes may play in low iron growth, both *mmar_1660* and *rv3041c* are in close genomic vicinity to another virulence hit in our screen, *fecB* (Figs 2C, 2E), a gene predicted to interact with *rv3041c* [56]. Interestingly, FecB (probable Fe^3+^-dicitrate-binding periplasmic lipoprotein) was recently found to bind the siderophore carboxymycobactin and to interact with known proteins of the (carboxy)mycobactin iron uptake pathway [80]. Arnold et al. have previously demonstrated that while the ABC transporter IrtAB imports both the hydrophilic carboxymycobactin and the hydrophobic mycobactin, it is essential only for mycobactin import, implying there is an additional yet-to-be-discovered importer for carboxymycobactin [81]. It is thus tempting to speculate that *mmar_1660*/*rv3041c*, together with *fecB* and/or other genes, is directly involved in transport of iron-laden carboxymycobactin into the bacterial cells.

We were not able to complement the low iron or virulence phenotypes of the Δ*1660* or *rv3041c* fs mutants, using a validated integrative plasmid (pFLAG_attP) with low-level constitutive expression of the genes of interest [82]. We speculate that this could be due to unbalanced expression and/or localization of *mmar_1660* and *rv3041c* in comparison to hitherto unknown codependent proteins for full function. To ensure that it was the introduced *mmar_1660* and *rv3041c* mutations that led to the observed phenotypes, we whole genome sequenced our strains. In Δ*1660* we observed only the introduced deletion, however, in the *rv3041c* fs we surprisingly found two additional SNPs that distinguished the mutant from the corresponding wt strain. One SNP in *lipB*/*rv2217*, encoding a probable lipoate biosynthesis protein B, changed an alanine (hydrophobic side chain) to glutamic acid (negatively charged side chain), potentially altering the protein’s function. *rv2217* is essential for *in vitro* growth according to several genome-wide studies [41,49,83]. Since our mutant grew comparably to the wt under normal iron *in vitro* conditions (Fig 4C) the introduced SNP clearly did not knock out a growth-essential mechanism of the encoded protein, making it less likely that the SNP made crucial changes to the gene’s function in virulence and low iron. The other SNP was found in *cut4*/*rv3452*, encoding a probable cutinase precursor, and changed an asparagine to a threonine, both amino acids with polar uncharged side chains. While *rv3452* is predicted to be non-essential for *in vitro* growth [41,49,83], in line with our observed *rv3041c* fs mutant normal iron phenotype, the similar properties of asparagine and threonine reduce the risk of this SNP to alter the function of the gene. These observations, strengthened by the lack of similar orthologous SNPs found in the *Mmar* Δ*1660* mutant, make us confident that our observed phenotypes were caused by the introduced mutations. Our ability to reproduce the same iron- and virulence-related phenotype by mutating the orthologous genes across two mycobacterial species further reinforces this confidence.

### Future perspectives

We used *Drosophila* to genome-widely identify mycobacterial virulence genes during host infection, complementing its role in studying innate immune responses towards mycobacterial infections. As a whole animal model, *Drosophila* hence provides a valuable platform for studying HPIs that affect mycobacterial disease outcomes, for instance by pinpointing specific genetic interactions between mycobacterial virulence factors and *Drosophila* host response mechanisms using TnSeq in combination with *Drosophila* host mutants. This capability was exemplified by Moule et al. in their Transposon Site Hybridization analysis of *Francisella novicida* genetic interaction with the *Drosophila* Imd signaling pathway [84]. Apparent limitations of the host model, such as the lack of adaptive immunity, may rather create an advantageous opportunity to specifically study HPIs relevant to innate immunity in an isolated yet *in vivo* setting. Of course, careful consideration in light of current available knowledge must be taken to assess the aptness of *Drosophila* to answer the individual research questions towards better understanding mycobacterial HPIs.

## Materials and methods

### Bacterial strains and growth conditions

The *Mycobacterium marinum* (*Mmar*) wt strains used in this study were *Mmar* E11 and *Mmar* M (NCBI GenBank accession numbers CP000854.1 and HG917972.2, respectively). The *Mycobacterium tuberculosis* (*Mtb*) strain was *Mtb* H37Rv (NCBI GenBank accession number NC_000962). The *Mmar* wt strains, in addition to the mutant strains *Mmar* E11 *eccB1::tn* (kanamycin resistant, [44]) and *Mmar* E11 dsRED (pSMT3dsRed, hygromycin resistant), were kind gifts from Wilbert Bitter at Vrije Universiteit Amsterdam. *Mmar* strains were cultured in Middlebrook 7H9 (Becton Dickinson, BD) supplemented with 0.2% glycerol, 0.05% Tween 80 and 10% ADC (50 g BSA fraction V, 20 g dextrose, 8.5 g NaCl, 0.03 g catalase, dH_2_O up to 1 L) for liquid growth. For solid growth, Middlebrook 7H10 (Becton Dickinson, BD) supplemented with 0.5% glycerol and 10% ADC or OADC (Becton Dickinson, BD), for *Mmar* or *Mtb*, respectively, was used. 20 μg/ml kanamycin, 50 μg/ml hygromycin, and 100 ng/ml anhydrotetracycline were added where required. *In vitro Mmar* growth curves were performed as previously described for *M. avium* [38], using the Bioscreen growth curve reader (Oy Growth Curves Ab Ltd.), with the exemption of temperature set at 30°C instead of 37°C. *Mtb* strains were cultivated as *Mmar* in Middlebrook 7H9 except for OADC (Becton Dickinson, BD) replacing ADC. *in vitro Mtb* growth curves were performed using Infinite M Plex microplate reader (TECAN). Experimental work using *Mtb* was carried out according to Norwegian regulation for organisms classified as Biosafely Level 3 (BSL3). The dedicated BSL3 laboratory is approved for BSL3 work with *Mtb* (Reference 12/8648–9 – Norwegian Directorate of Health) and is located within a restricted area for authorized personnel.

### Low iron growth conditions

*Mmar* and *Mtb* strains were cultured in Middlebrook 7H9 with supplements as described above until mid log phase. The strains were washed twice in low iron medium prepared as following: 5 g L-asparagine, 5 g KH_2_PO_4_, 0.25% glycerol, 0.05% Tween 80, 0.5 g BSA, and 0.2 g dextrose was dissolved in 900 ml H_2_O. The solution was adjusted to pH 6.8 with NaOH and H_2_O was added to reach 1000 ml. The solution was chelated with magnetic stirring for 1–3 days with 20–50 g Chelex 100 resin (Bio-Rad Laboratories) before filtered (0.2 μM pores) into a plastic bottle. Finally, 50 μl 10 mg/ml ZnCl_2_, 50 μl 2 mg/ml MnCl_2_ and 200 μl 200 mg/ml MgSO_4_, all prepared in iron free water in plastic containers and sterile filtered, was added. For *Mtb* growth curves, the strains were subcultured once (after wash) to mid log phase before back-diluted to OD_600_ 0.02. Deferoxamine (DFO, Sigma Aldrich) was furthermore added to the low iron medium at a final concentration of 1 μM. For *Mmar*, the strains were directly back-diluted to OD_600_ 0.02 for growth curve experiments. FeCl_3_ was added to the high iron growth curves to a final concentration of 150 μM for *Mmar* and 300 μM for *Mtb*.

### *Mmar_1660* knockout strain

To create the *mmar_1660* knockout strain (Δ*1660*), bacteriophage-mediated allelic exchange using pYUB1471 and phAE159 was used (kind gift from William R Jacobs at Albert Einstein College of Medicine), following published protocols [85]. In short, around 1000 bp upstream and downstream of *mmar_1660* were amplified, and cloned into pYUB1471, creating pYUB_1660. pYUB_1660 was further cloned into phAE159, which was used to transduce *Mmar* M to induce allelic exchange in order to replace the *mmar_1660* gene with genes encoding hygromycin resistance (hygR) and levansucrase (sacB) genes. Positive clones were selected on 7H10 agar containing hygromycin and further validated by PCR.

### *Mtb rv3041c* fs knockout strain

To create the *rv3041c* knockout strain (*rv3041c* fs), sgRNA targeting the gene was cloned into pCRISPRx-Sth1Cas9-L5 (addgene plasmid #140993) using *Bsm*BI restriction sites and oligonucleotides aaacaacgtgtccctgcgccgtaat and gggaattacggcgcagggacacgtt according to published protocols [86] creating pCRISPR_*3041c*. pCRISPR_*3041c* was subsequently transformed into *Mtb* H37Rv wt and selected on 7H10 agar plates containing 20 μg/ml kanamycin. After incubation at 37°C for 3–4 weeks, colonies were re-streaked on 7H10 agar plates containing 100 ng/ml anhydrotetracycline and incubated 37°C for 3–4 weeks. Arising colonies were analyzed for *rv3041c* fs mutations by PCR against *rv3041c* using oligonucleotides gttgggcgtgactcgcgg and acgctgatccggcgtccg. PCR products were purified using AMPure XP beads (Beckman Coulter) and sent for Sanger sequencing using the gttgggcgtgactcgcgg oligonucleotide. Mutations were identified using Synthego ICE (Synthego Performance Analysis, ICE Analysis. 2019. v3.0. Synthego). The selected mutant had a 5 base pair deletion within the *rv3041c* gene, creating a fs and a subsequent early stop codon after 30 amino acids (the full length *rv3041c* gene product is 288 amino acids).

### Whole genome sequencing

Genomic DNA was prepared using Epicentre MasterPure Complete DNA Purification kit. Sequencing libraries were prepared using the Illumina DNA prep kit and sequenced on an Illumina MiSeq instrument using the MiSeq Reagent Kit v3 with 300 bp paired end configuration. Raw data were quality controlled using FastQC v0.11.9 [87]. Reads were mapped, filtered and variants called relative to the respective reference genomes (CP000854.1 for *Mmar* M strain and NC_000962.3 for *Mtb* H37Rv) using Snippy v4.4.3 [88]. Average coverage for each sample was calculated using Samtools v1.9 [89]. Large deletions were additionally identifed by defining regions with zero coverage using Geneious Prime v2023.0.4 (https://www.geneious.com). For *Mtb* strains, variants were filtered using tb_variant_filter v0.4.0, excluding PE/PPE genes [90], UVP repetitive loci and Refined Low Confidence (RLC) plus low mappability regions. For *Mmar* strains, all CDS annotated as PE/PPE-family proteins were excluded. For *Mtb* and *Mmar* knockout strains, variants that were also detected in wt or in very low coverage regions (<10x) of the wt strain were disregarded. WGS sequence data are available from the NCBI Sequence Read Archive under BioProject PRJNA1068332.

### *Mmar* tn mutant library

The high-density tn mutant *Mmar* E11 library was prepared as previously described for *M. avium* [38]*,* using ϕMycoMarT7 (kind gift from Eric Rubin at Harvard T.H. Chan School of Public Health [47]), except for heating medium and bacterial cultures to 30°C as opposed to 37°C prior to transduction, and incubating the library at 30°C as opposed to 37°C on 7H10 plates with Tween 80 (0.05%) and kanamycin (20 μg/ml) for 2 weeks.

### Bacterial single-cell suspension for infection

*Mmar* strains and the *Mmar* E11 tn mutant library were prepared to obtain single-cell suspensions prior to *Drosophila* infections. Strains were cultured to stationary phase, pelleted down by centrifugation, and resuspended in 7H9 medium with 0.2% Tween 80 to resolve larger clumps. Cultures were then pelleted again and resuspended in 7H9 media with 15% glycerol. The resuspension sat at room temperature for 30 minutes to let clumps fall to the bottom before the supernatant was transferred and aliquoted to cryotubes for storage at -80˚C. To calculate CFU/mL of the infection stocks, they were diluted and spotted on 7H10 agar plates to determine the CFU/mL for *Drosophila* infection. For iPSDM infection, *Mtb* and *Mmar* M strains were prepared to obtain singe-cell suspension at the day of macrophage infection. Mid-log cultures, pelleted down and resuspended in RPMI 1640 with 10% FCS, underwent vortexing (2 times for 30 s), sonication at the power of 70% (2 times for 5 s), and vortexing 2 times for 30 s again. The cultures were centrifuged at 300 g for 4 min as a final clump-removing step. The CFU/mL of *Mtb*-containing supernatants used to infect cells were calculated by an OD_600-_conversion of 1 = 4.5 x 10^8^ CFU/mL). The CFU/mL of *Mmar*-containing supernatants used to infect cells were calculated by an OD_600-_conversion of OD_600_ 1 = 2 x 10^7^ CFU/mL.

### *Drosophila* strains

The *Drosophila melanogaster* (*Drosophila*) strain used for TnSeq was a cross between the Transgenic RNAi Project (TRiP) control fly AttP40 (y v; attP40, y + , stock #36304 at the Bloomington *Drosophila* Stock Center, BDSC) and a tubulin-Gal4 driver fly. For all other *Drosophila* infections, the Oregon R-C (Flybase ID FBsn0000276, stock #5 at the BDSC) was used. Flies were bread at 25ºC with constant light:dark cycles of 12 hours each and a humidity of 70%. The Bloomington standard cornmeal formulation containing yellow cornmeal, corn syrup solids, inactive nutritional yeast, agar and soy flour was used to feed flies (pre-mixed dry version available at Genesee Scientific).

### *Drosophila* infection, survival and CFU assay

Infections were performed in 3–5-days-old flies. Anaesthetized flies were infected into the abdomen using a Nanoject II (Drummond Scientific Company) set to inject 13.8 nl using glass needles prepared using a PB-7 needle puller (NARISHIGE) and were not exposed to CO_2_ anesthesia for more than 15 minutes during the process. Bacterial infection stocks were prepared as previously described [25] and diluted to 500 or 5000 CFU/13.8 nl in 1:1 ratio of phosphate-buffered saline (PBS) to Brilliant Blue (Sigma Aldrich). Flies were incubated at 29°C after infection. For CFU assays, flies were put briefly into 70% ethanol before washed once with PBS and homogenized in 100 μl PBS using a pestle. The homogenates were spotted in a 10-fold dilution series on 7H10 agar plates containing 20 μg/ml kanamycin and 1.25 μg/mL Amphotericin B (to eliminate the fly’s microbiota). For each CFU count, three flies per condition were harvested, homogenized and treated separately during dilutions and spotting. Two technical replica per fly were spotted. The agar plates were grown for around 10 days at 30°C before counting CFUs. For TnSeq, 250 flies were infected with 5000 CFU of *Mmar* E11 tn library per fly in three biological replica (3 x 250 infected flies). 10 and 10 flies were put briefly into 70% ethanol before washed once with PBS and homogenized in 200 ul PBS using a pestle. The homogenates from 10 and 10 flies were plated onto 15 cm diameter 7H10 agar plates containing 20 μg/ml kanamycin, 1.25 μg/mL Amphotericin B and 0.05% Tween 80, ending up with around 25 plates per library, and grown for 14 days at 30°C. The three biological replicas were treated separately throughout the experiment. For survival assays, 30 flies were infected per condition and the number of dead and live flies was noted every morning.

### Transposon insertion sequencing, TnSeq

The tn library was harvested and pooled by scraping agar plates with colonies. Total DNA was purified using Masterpure DNA purification kit (Epicentre) and prepared for TnSeq by PCR amplification of tn-genome junctions and adapter ligation as previously described [91]. The samples were sequenced on an Illumina NextSeq 2000, generating around 12–15 million 150 + 150 bp paired-end reads per sample.

### Bioinformatic analysis of TnSeq datasets

The reads were processed using TPP in TRANSIT [92], which counts reads mapping to each TA dinucleotide site. Beta-Geometric correction was applied to the datasets to adjust for skewness [93]. Essential genes were identified using a hidden Markov model (HMM), incorporated into TRANSIT [92], as described in more detail previously for *M. avium* [38]. Virulence genes (comparative analysis between input and output tn libraries; determining statistical differences in sum of tn insertion counts in genes within library selected *in vitro* versus after infection) were identified using the “resampling” algorithm incorporated into TRANSIT [92].

### Macrophage infection and CFU assay

Human induced pluripotent stem cell (iPSC) were obtained from European Bank for induced pluripotent Stem Cells (EBiSC, https://ebisc.org/about/bank), distributed by the European Cell Culture Collection of Public Health England (Department of Health, UK) and produced into monocytes as previously described for our laboratory [94], based on the protocol of Armesilla-Dias et al. [95]. Monocytes were seeded in 96-well plates (40 000/well) and differentiated into macrophages in RPMI 1640 with 10% fetal calf serum (FCS) and 100 ng/mL M-CSF (Prepotech, 300–25). At day five of differentiation, the cells’ medium was changed to RPMI 1640 with 10% FCS (normal iron) or RPMI 1640 with 10% FCS with 100 μM ferric ammonium citrate (FeAC) (high iron). At day 6 of differentiation the cells were infected with the respective *Mmar* strains at a multiplicity of infection of 4:1 macrophage:bacterium followed by incubation at 33˚C and 5% CO_2_. After one hour incubation, the cells were washed once with phosphate-buffered saline (PBS) to remove extracellular bacteria before adding RPMI 1640 with 10% FCS or RPMI 1640 with 10% FCS with FeAC. At day 0 and 1 post infection cells were washed twice with Hanks balanced salt solution before being lysed in 100 μl PBS with 0.1% Triton X-100 (Sigma-Aldrich). The cell lysate was then spotted in a 10-fold dilution series on 7H10 agar plates containing 10% ADC and 0.2% glycerol. The plates were incubated at 30˚C and taken out when CFUs appeared in dilutions with optimal countable range. Macrophage infection with *Mtb* strains were conducted in a similar manner to *Mmar* strains except for a few key differences. *Mtb*-infected macrophages and bacteria-containing agar plates, the latter supplemented with OADC instead of ADC, were kept at 37˚C instead of 30˚C. *Mtb* proliferates at a slower rate than *Mmar*, hence, cells were lysed at day 0, 3 and 6 post infection to determine intracellular bacterial growth. Due to the extended time-period of the experiment, half-volume media change was carried out on day 3 post infection.

## Supporting information

S1 Fig Correlation of the gene-level mean tn insertion counts among independent biological replicates. Scatter plot matrices showing the correlation of the gene-level mean insertion counts among the three replicates of the conditions *in vitro* input library (*in vitro* 1–3), female flies (AttP40_F 1–3), and male flies (AttP40_M 1–3). The fourth matrix shows the correlation among the three different conditions, where gene means for each condition are averaged over the three replicates.
(PDF)

S1 Dataset TnSeq analysis of *Mmar* tn libraries. (A) Essential gene analysis: *Mmar* E11 *in vitro* genetic requirement (determined by HMM in TRANSIT [92]). (B) Virulence gene analysis: *Mmar* E11 genes required for infection in male *Drosophila* (determined by resampling analysis in TRANSIT [92]) = log fold change >0 and adjusted P-value <0.05. (C) *Mmar* E11 genes required for infection in male versus female *Drosophila* (determined by resampling analysis in TRANSIT [92]) = log fold change >0 and adjusted P-value <0.05.
(XLSX)

S2 Dataset WGS of *Mmar* wt, Δ*1660* mutant, *Mtb* wt, and *rv3041c* fs mutant strains. (A) Variants identified in *Mtb* wt and knockout strains. (B) Zero coverage regions identified in *Mtb* wt and knockout strains. (C) Variants identified in *Mmar* wt and knockout strains. (D) Zero coverage regions identified in *Mmar* wt and knockout strains.
(XLSX)
