# Supplementary material for: Genome-wide phenotypic insights into mycobacterial virulence using Drosophila melanogaster
Source: PLoS Pathog. 2025 Sep 5;21(9):e1013474. doi: 10.1371/journal.ppat.1013474 (PMC12425279; doi:10.1371/journal.ppat.1013474)
Supplement: S1 Fig — Scatter plot matrices showing the correlation of the gene-level mean insertion counts among the three replicates of the conditions in vitro input library (in vitro 1–3), female flies (AttP40_F 1–3), and male flies (AttP40_M 1–3). The fourth matrix shows the correlation among the three different conditions, where gene means for each condition are averaged over the three replicates. (PDF) [file ppat.1013474.s001.pdf]

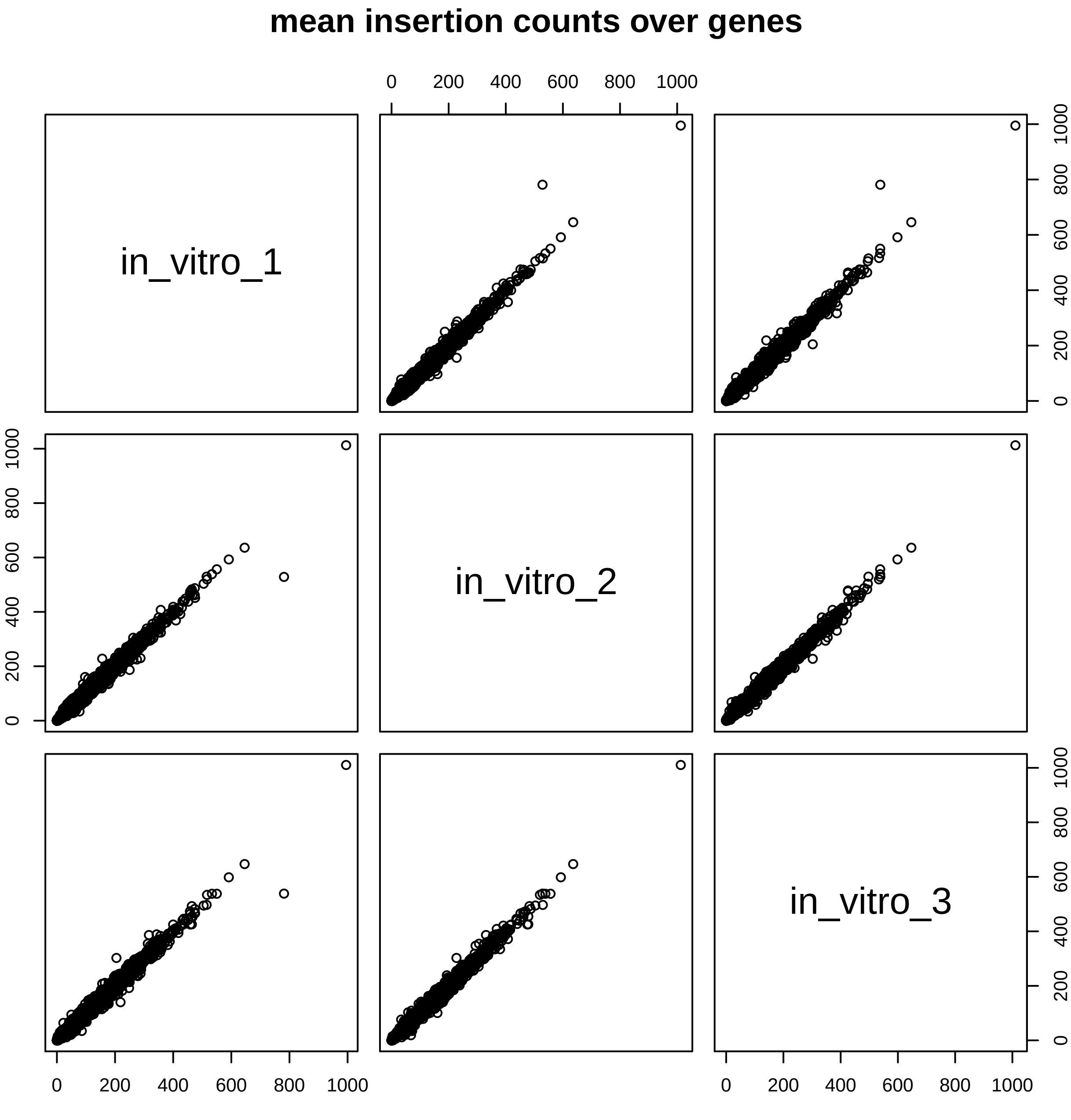
mean insertion counts over genes
in_vitro_1
in_vitro_2
in_vitro_3
0
200
400
600
800
1000

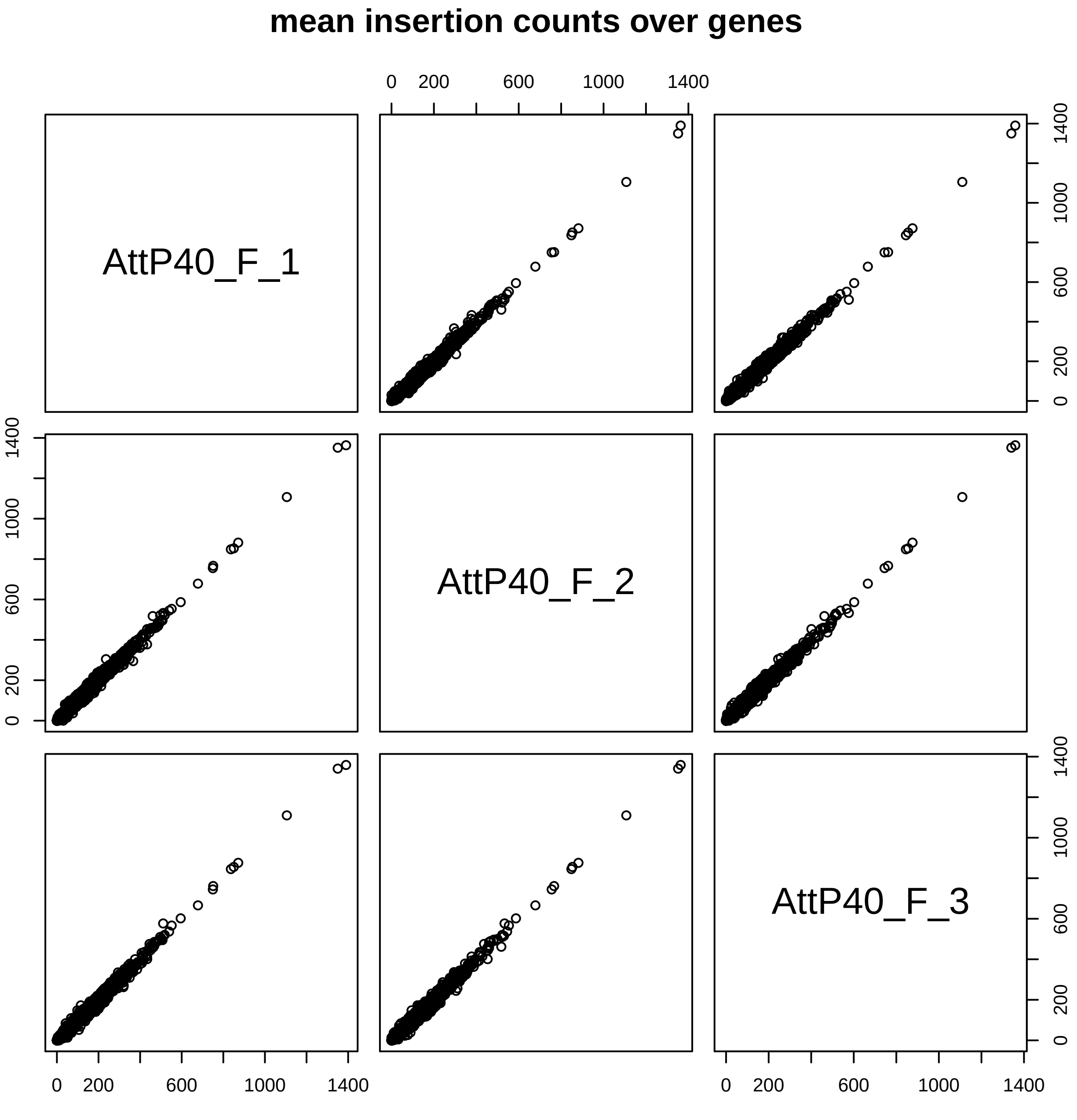
mean insertion counts over genes
AttP40_F_1
AttP40_F_2
AttP40_F_3
0
200
600
1000
1400

mean insertion counts over genes

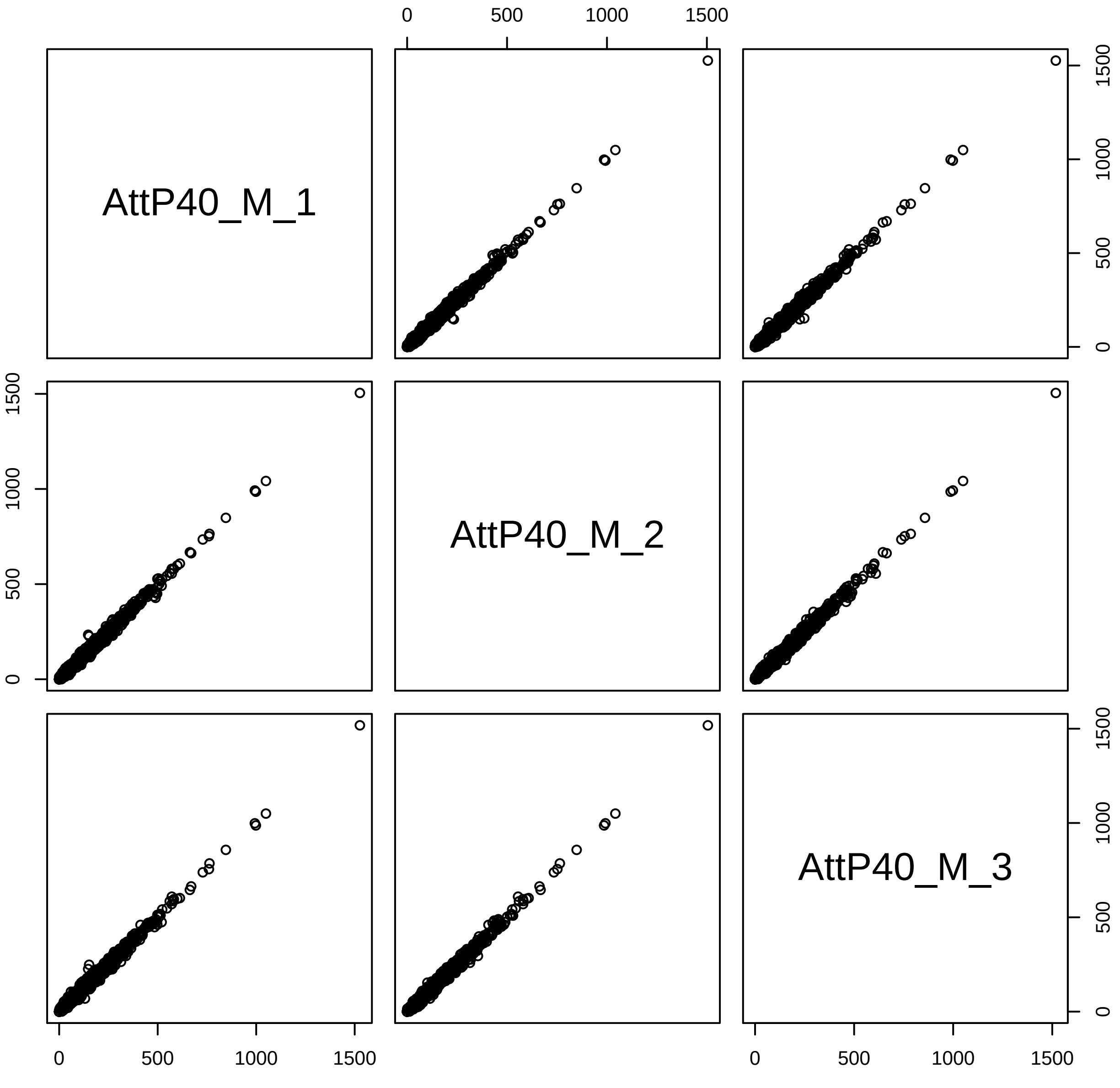

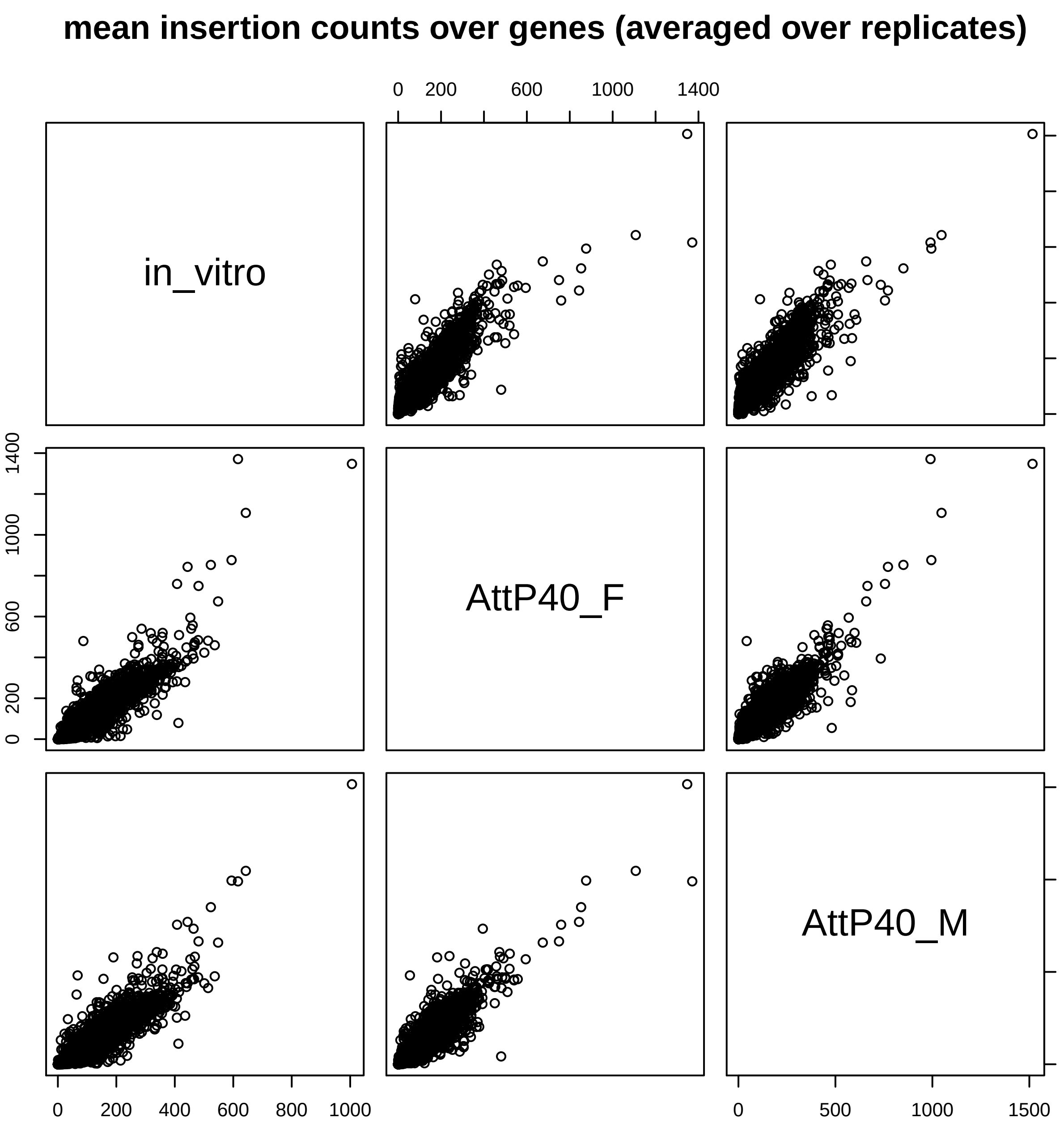
mean insertion counts over genes (averaged over replicates)
in_vitro
AttP40_F
AttP40_M
0
200
600
1000
1400
0
200
400
600
800
1000
0
200
600
1000
1400
0
500
1000
1500
0
200
400
600
800
1000
0
500
1000
1500
